# Crude leaf extracts of Piperaceae species downmodulate inflammatory responses by human monocytes

**DOI:** 10.1371/journal.pone.0198682

**Published:** 2018-06-20

**Authors:** Angela Carolina Finato, Thais Fernanda Fraga-Silva, Amanda Uliana Carvalho Prati, Amauri Alves de Souza Júnior, Bruna Fonseca Mazzeu, Lidiane Gaspareto Felippe, Rute Alves Pinto, Marjorie de Assis Golim, Maria Sueli Parreira Arruda, Maysa Furlan, James Venturini

**Affiliations:** 1 Universidade Estadual Paulista (Unesp), Faculdade de Ciências, Bauru, SP, Brazil; 2 Universidade Estadual Paulista (Unesp), Instituto de Biociências, Botucatu, SP, Brazil; 3 Universidade Estadual Paulista (Unesp), Instituto de Química, Araraquara, SP, Brazil; 4 Universidade Estadual Paulista (Unesp), Faculdade de Medicina, Botucatu, SP, Brazil; 5 Universidade Federal de Mato Grosso do Sul (UFMS), Faculdade de Medicina, Campo Grande, MS, Brazil; National Institute of Allergy and Infectious Diseases, UNITED STATES

## Abstract

In this study, we aimed to evaluate the immunomodulatory effects of crude leaf extracts from *Piper gaudichaudianum* Kunth, *P*. *arboreum* Aub., *P*. *umbellata* L., *P*. *fuligineum* Kunth, and *Peperomia obtusifolia* A. Dietr. on an *in vitro* model of inflammatory response. The crude extracts were previously obtained by maceration of the leaves. The half-maximal inhibitory concentration was determined by the MTT assay using human peripheral blood mononuclear cells. Human monocytes were simultaneously challenged with each crude extract and lipopolysaccharide (LPS), the major component of the outer membrane of Gram-negative bacteria, to induce a strong inflammatory response. After 24 h of incubation, cell-free supernatants were used for evaluating the mediators involved in inflammation: H_2_O_2_, TNF-α, IL-8, IL-6, IL-1β, IL-10, IL-12, FGF-b, and TGF-β1. We also compared the results with the effects of ketoprofen, a well-known anti-inflammatory drug. The *P*. *gaudichaudianum* crude extract downmodulated the production of H_2_O_2_, IL-1β, IL-6, IL-8, and TGF-β1 by LPS-stimulated monocytes; *P*. *arboreum*, IL-1β, IL-6, IL-8, and TNF-α; *P*. *umbellata* and *P*. *fuligineum*, H_2_O_2_, IL-1β, IL-6, IL-8, IL-10, and TNF-α; and *P*. *obtusifolia*, H_2_O_2_, IL-6, IL-8, IL-10, and TNF-α. In general, the crude leaf extracts amplified the anti-inflammatory response when compared with ketoprofen, particularly reducing the production of IL-8, a mediator involved in neutrophil recruitment during tissue damage. Thus, the crude leaf extracts of *P*. *gaudichaudianum*, *P*. *arboreum*, *P*. *umbellata*, *P*. *fuligineum*, and *Peperomia obtusifolia* elicited an anti-inflammatory response against LPS-challenged monocytes. These findings show the anti-inflammatory properties of these crude leaf extracts and offer new perspectives for their use in the treatment of inflammatory diseases.

## Introduction

The family Piperaceae comprises pantropical herbal plants, widely distributed in Latin America, particularly from Mexico to the southwest of Argentina [1–3]. The family is composed of five genera: *Piper*, *Peperomia*, *Manekia*, *Zippelia*, and *Verhuellia* [4,5]. *Piper* and *Peperomia* are the most representative genera with 2000 and 1700 species, respectively [6,7]. Besides their economic importance, Piperaceae species have been used in traditional medicine as an anti-inflammatory agent, for relief from toothache, gynecological illnesses, and intestinal disorders, as well as psychotropic and anxiolytic agents [8]. *Piper* and *Peperomia* species possess various classes of bioactive compounds, such as amides [9,10], lignans [11,12], secolignans [13,14], phenylpropanoids [15], prenylated benzoic acid derivatives [16], chromenes and chromanes [17–21], terpenes [22], alkaloids [23–25], and others [26,27].

Previous chemical and biological studies on Piperaceae have revealed them to be a rich source of new biologically active secondary metabolites. The accumulation of a major secondary metabolite, 4-nerolidylcatechol, was observed in *P*. *umbellata* [syn. *Pothomorphe umbellata* (L.) Miq., *Heckeria umbellata* (L.) Kunth., *Piper hilarianum* Stend] leaves [28]. The metabolite exhibits potent anti-oxidant and anti-inflammatory activities [29]. The potent inhibitory effect of kavalactones in *P*. *fuligineum* against hepatitis C virus replication was recently described [30]. Their potential anti-inflammatory and anxiolytic properties were also described [31]. *P*. *arboreum* possesses antifungal, trypanocidal, antimicrobial, and anti-oxidant pyrrolidine amides as major natural compounds [9,32–34]. The accumulation of prenylated chromenes and chromanes with potent trypanocidal activity against the Y-strain of *Trypanosoma cruzi* was observed in *P*. *gaudichaudianum* and *Peperomia obtusifolia* [18,20,21]. Chemical studies with *P*. *gaudichaudianum* demonstrated the presence of gaudichaudianic acid, a prenylated chromene that is a major secondary metabolite in leaves and roots of this species [18,19]. In terms of biological activities, this compound showed potent trypanocidal and antifungal activities against plant pathogens. Furthermore, the unusual presence of two natural isomeric forms of gaudichaudianic acid [(+)-*S* and (-)-*R*] was observed during the isolation of such compounds, as well as their synergistic effect, with the racemic mixture being the most active in trypanocidal assays [20].

In this study, we revealed new biological properties of the crude leaf extracts of *P*. *gaudichaudianum* Kunth, *P*. *arboreum* Aub., *P*. *umbellata* L., *P*. *fuligineum* Kunth, and *Peperomia obtusifolia* A. Dietr., by characterizing their immunomodulatory effects on an *in vitro* model of human inflammatory response.

## Material and methods

### Plant material

*P*. *gaudichaudianum* leaves were collected from the campus of the University of São Paulo, Brazil, and identified by Dr. Inês Cordeiro (Botanic Garden curator of University of São Paulo, Brazil). A voucher specimen (Kato-0093) has been deposited at the Herbarium of the Botanic Institute, São Paulo, Brazil. *P*. *arboretum*, *P*. *umbellate*, and *Peperomia obtusifolia* leaves were collected from the greenhouse of the Institute of Chemistry of UNESP, Araraquara, SP, Brazil, and identified by Dr. Inês Cordeiro and Dr. G. E. D. Paredes (University of Pedro Ruiz Gallo, Peru), respectively. Voucher specimens [(Cordeiro-1936), (Kato-671), (Kato-070), respectively] have been deposited at the Herbarium of the Botanic Institute of University of São Paulo, Brazil. *P*. *fuligineum* leaves were collected from the Botanic Garden, Araraquara, São Paulo, Brazil, and identified by Dr. Inês Cordeiro. A voucher specimen (Kato-0720) has been deposited at the Herbarium of the Botanic Garden of the University of São Paulo, Brazil.

### Preparation of crude extracts

The preparation of the ethanolic extracts and chemical characterizations of *P*. *gaudichaudianum*, *P*. *arboreum*, and *P*. *umbellata* have been previously described [29,35,36]. Briefly, leaves of these species were milled, extracted with ethanol (EtOH), and the extract concentrated under vacuum to yield the crude extracts.

Dried leaves of the *P*. *fuligineum* were milled and extracted with ethanol (EtOH). This ethanolic extract was concentrated under vacuum to obtain 54.4 g of the concentrate, which was resuspended in MeOH:H2O (4:1) and partitioned with hexane, CHCl3, and EtOAc successively. The soluble CHCl3 fraction (13 g) was subjected to bioassay. Dried leaves (430 g) of *Peperomia obtusifolia* were milled and extracted by maceration at room temperature with EtOAc (3 × 1000 mL) for 72 h. The resulting solution was filtered and concentrated under reduced pressure to obtain 21 g of crude extract. The EtOAc extract was subjected to bioassay. Final stock concentrations of extracts were 100 mg/mL.

### Experimental design

The study was performed in two steps. First, we evaluated the cytotoxicity and inhibitory concentration of 50% (IC_50_) of each crude extract using peripheral blood mononuclear cells (PBMCs). Then, we evaluated the immunomodulatory properties of the five Piperaceae species using a widely known *in vitro* model for lipopolysaccharide (LPS)-mediated inflammatory response. LPS is an endotoxin of Gram-negative bacteria that triggers an intense release of pro-inflammatory cytokines by monocytes. For each assay, cells were stimulated with ketoprofen, an anti-inflammatory drug. Human monocytes were cultivated and subjected to six treatments: (1) medium (unstimulated control), (2) LPS, (3) crude extracts **(***P*. *gaudichaudianum*, *P*. *arboreum*, *P*. *umbellata*, *P*. *fuligineum* and *Peperomia obtusifolia***)**, (4) LPS + crude extracts, (5) ketoprofen, and (6) LPS + ketoprofen. Each assay was performed in duplicate or triplicate.

### Isolation of PBMCs

Human peripheral venous blood was obtained from six healthy donors. The blood was collected with Vacutainer® tubes containing heparin as an anticoagulant. PBMCs were isolated by density gradient centrifugation on Histopaque^®^-1077 (Sigma-Aldrich, St Louis, MO, USA). PBMCs were centrifuged and resuspended in 1.0 mL of RPMI-1640 (Nutricell, Campinas, SP, Brazil) supplemented with 20% heat-inactivated fetal calf serum (FCS) (Gibco BRL, Grand Island, NY, USA), penicillin (100 UI mL), and streptomycin (100 mg mL) (Gibco). Cell viability, as determined by 0.2% trypan blue, was >95% in all experiments. Concentration was adjusted to 1.0 × 10^6^ cells/mL using Turk stain.

### Cytotoxicity and IC_50_

Cytotoxic activity of the crude extracts was determined by the colorimetric microculture 3-(4,5-dimethyl-2-thiazolyl)-2,5-diphenyl-2H-tetrazolium bromide (MTT) assay [37]. PBMCs (1.0 × 10^5^ / well) were seeded into 96-well flat bottom plates, cultured in the presence of each crude extract dissolved in dimethyl sulfoxide (DMSO), and serially diluted in phosphate buffered solution (PBS) (0.156, 0.313, 0.625, 1.25, 2.5, 5.0 mg/mL). The final concentration of DMSO at each crude extract was always less than 1%. Concanavalin A (10 mg/mL), a potent mitogen for T lymphocytes, was used as the internal control for lymphoproliferation. After continuous incubation for 96 h at 37°C under 5% CO_2_ atmosphere, the culture plate was centrifuged for 5 min at 1500 rpm and supernatants were replaced with 20.0 μL of MTT solution (5.0 mg/mL) plus 100.0 μL of supplemented RPMI-1640 medium. After incubation for 2 h, the supernatant was removed, and formazan crystals that formed in viable cells were dissolved in 100.0 μL of DMSO per well. The optical densities were measured using an ELISA microreader (EL800, BIO-TEK Instruments, INC) at a wavelength of 540 nm. The cytotoxic index was determined by the ratio between treated cells and non-treated cells. The IC_50_ was determined by linear regression analysis.

### Monocyte cell culture

Mononuclear cells were obtained as previously described. To obtain human monocytes, mononuclear cells were counted and adjusted to 1.0 × 10^6^ of mononuclear phagocytes/mL. The viability was higher than 90%, as judged by the uptake of 0.02% neutral red (Sigma-Aldrich). The cells were distributed in a volume of 100 μL per well into 96-well flat bottom plates and incubated for 2 h at 37°C in a humid atmosphere with 5% CO_2_ to allow monocytes to adhere. Non-adherent cells were removed by washing the wells thrice with RPMI-1640 supplemented with 10% FCS, penicillin (100 UI mL) and streptomycin (100 mg mL) and the remaining monocytes (>90% mononuclear phagocytes as assessed by morphological examination and expression of CD14, CD19 and CD3 by fluorescence-activated cell sorting) were used for experiments. In order to evaluate the influence of compounds in an inflammatory environment, we next subjected monocytes to six treatments, as following: (1) medium (unstimulated control), (2) LPS– 10 μg/mL, (3) each crude extracts at concentration of IC_50_, (4) LPS + crude extracts, (5) ketoprofen– 5.2 mM [38], and (6) LPS + ketoprofen. All the substances and compounds were added simultaneously. Culture cells were incubated at 37°C under 5% CO_2_ atmosphere for 24 h. At the end of the cell culture period, the supernatants were removed and stored at– 80°C for determination of cytokines and the hydrogen peroxide release assay.

### Hydrogen peroxide release

At the end of the cell culture period, the monocytes were incubated with phenol red solution [dextrose 1% (Sigma-Aldrich), phenol red 1% (Sigma-Aldrich), horseradish peroxidase type II– 5 UI (Sigma-Aldrich)] and plated at 37°C under 5% CO_2_ atmosphere for 1 h according to the method described by Russo *et al* [39]. The reaction was stopped by the addition of 1.0 N NaOH and the H_2_O_2_ concentration was determined using an ELISA microreader at 620 nm.

### Cytokine analysis

The levels of interleukin (IL)-8, IL-1β, IL-6, IL-10, TNF-α, and IL-12 p70 were determined by flow cytometry using a BD cytometric bead array (CBA) (BD Biosciences, CA, USA). The levels of transforming growth factor beta (TGF-β) and basic fibroblast growth factor (basic FGF) were determined by ELISA using a cytokine Duo-Set Kit (R&D Systems, Minneapolis, MN, USA), according to the manufacturer’s instructions.

### Statistical analysis

All experimental protocols were performed at least three times. The results of the controls (medium and ketoprofin, unstimulated and LPS stimulated) were used in the analyses for all extract evaluated. Comparison of the treatments was performed by repeated measures ANOVA with Bonferroni post-test. The analyses were conducted using GraphPad Prism 5.0 (GraphPad Software. Inc., San Diego, CA, USA) and statistical significance was set at p-value < 0.05.

### Ethics Committee

This study was approved by the Research Ethics Committee of the Faculdade de Ciências de Bauru—UNESP (Certificate of Presentation for Ethical Consideration–CAAE #20497414.0.0000.5398). Written informed consent to participate and publish the data was obtained and signed by all the participants.

## Results

Table 1 presents the IC_50_ for each crude extract. The values ranged from 0.24 mg/mL to 2.19 mg/mL. The complete results of the cytotoxicity assay are shown in S1 Fig.

**Table 1:** IC_50_ values of the crude extracts of *P*. *gaudichaudianum*, *P*. *arboreum*, *P*. *umbellata*, *P*. *fuligineum* and *Peperomia obtusifolia*.

*Crude extracts*	IC_50_ (mg/mL)
*P*. *gaudichaudianum*	0.55
*P*. *arboreum*	2.19
*P*. *umbellata*	1.56
*P*. *fuligineum*	0.24
*Peperomia obtusifolia*	2.12

**Fig 1:**
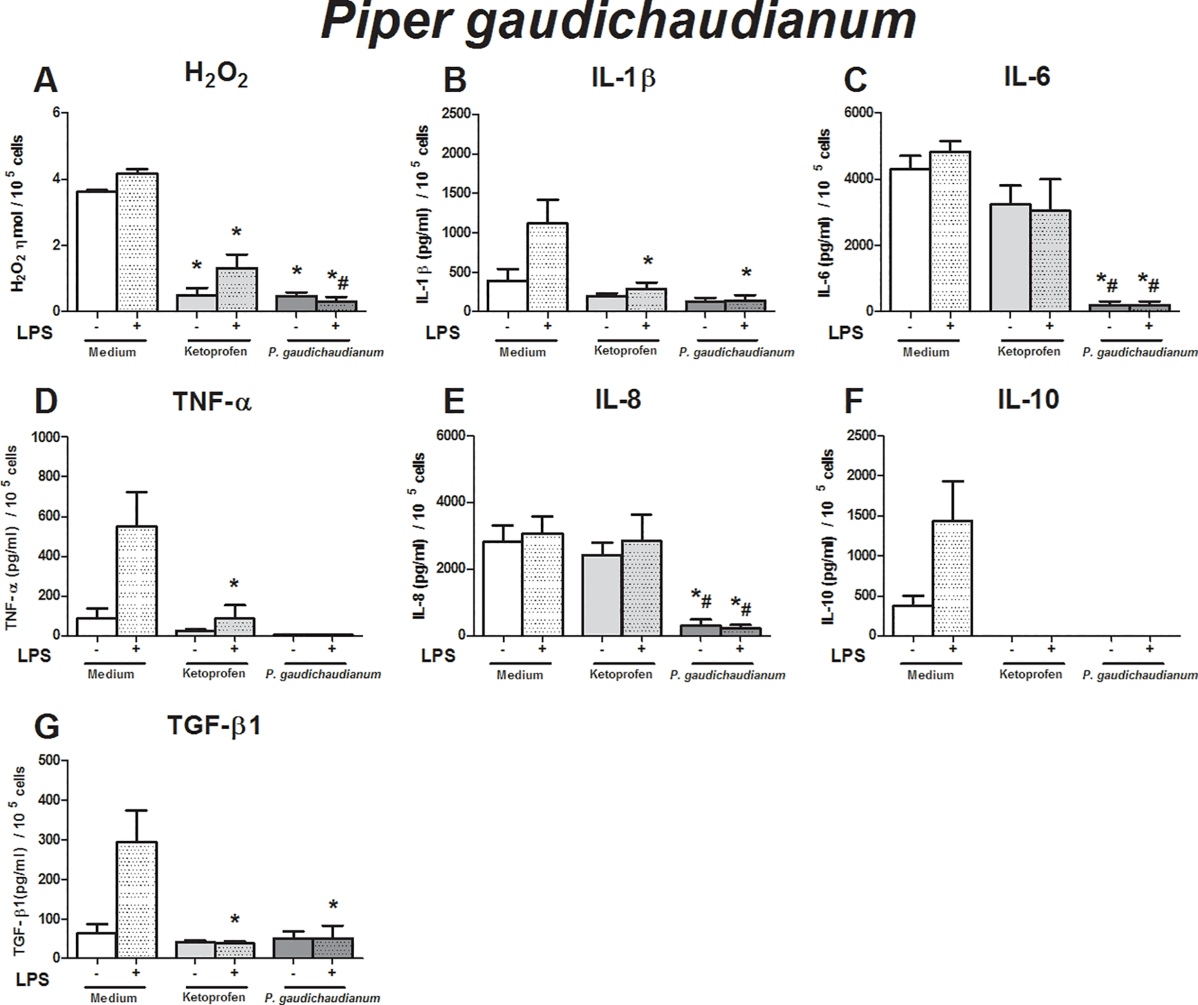
Immunomodulatory effect of crude leaf extract of *Piper gaudichaudianum* on LPS-stimulated human monocytes. The results of the controls (medium and ketoprofin, unstimulated and LPS stimulated) were used in the analyses for all extract evaluated. Data are expressed as median ± SEM (pg/mL). ANOVA with Bonferroni post-test; p < 0.05; *: significantly different from placebo treatment, #: significantly different from ketoprofen treatment.

**Fig 2:**
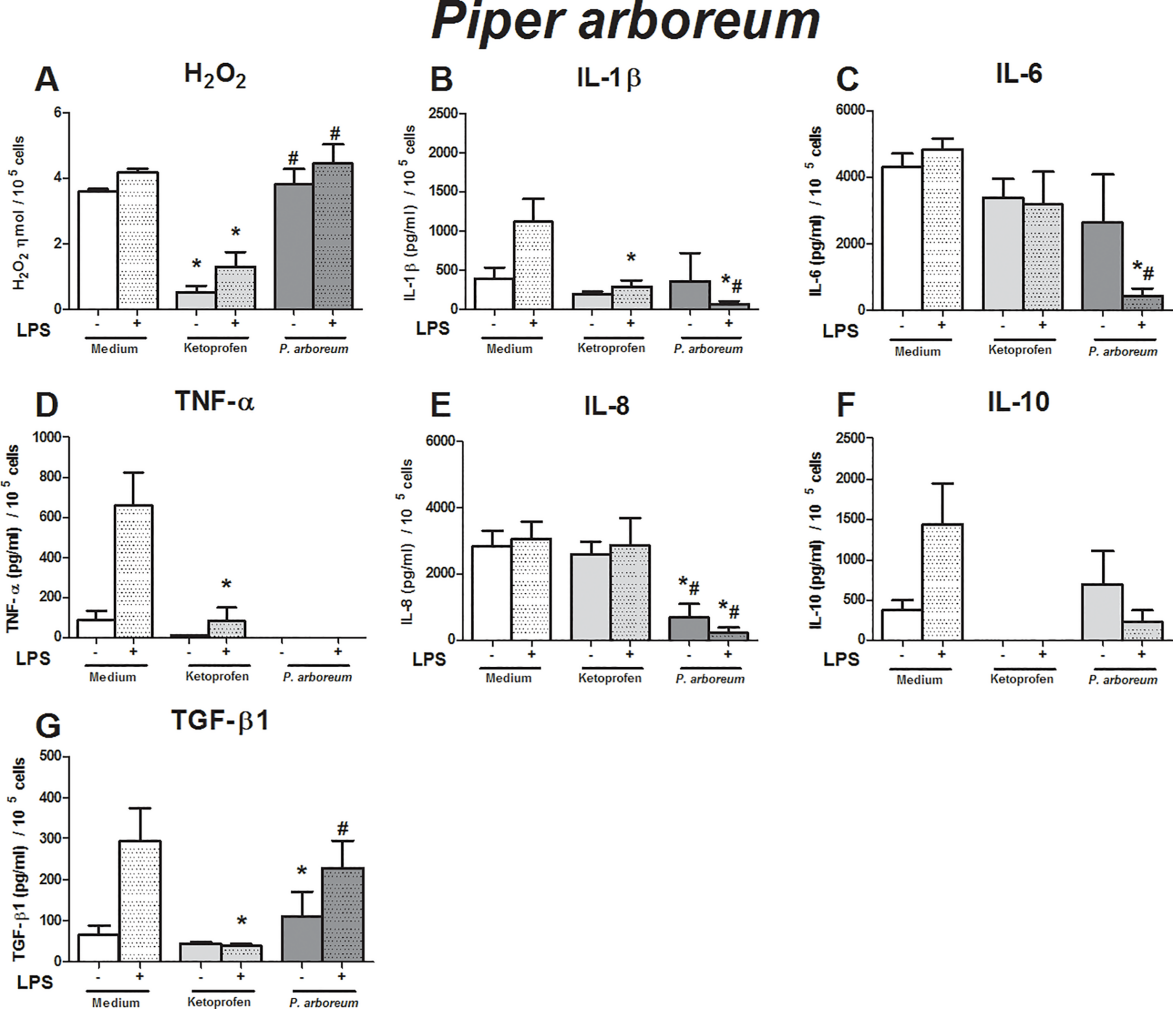
Immunomodulatory effect of crude leaf extract of *Piper arboreum* on LPS-stimulated human monocytes. The results of the controls (medium and ketoprofin, unstimulated and LPS stimulated) were used in the analyses for all extract evaluated. Data are expressed as median ± SEM (pg/mL). ANOVA with Bonferroni post-test; p < 0.05; *: significantly different from placebo treatment, #: significantly different from ketoprofen treatment.

**Fig 3:**
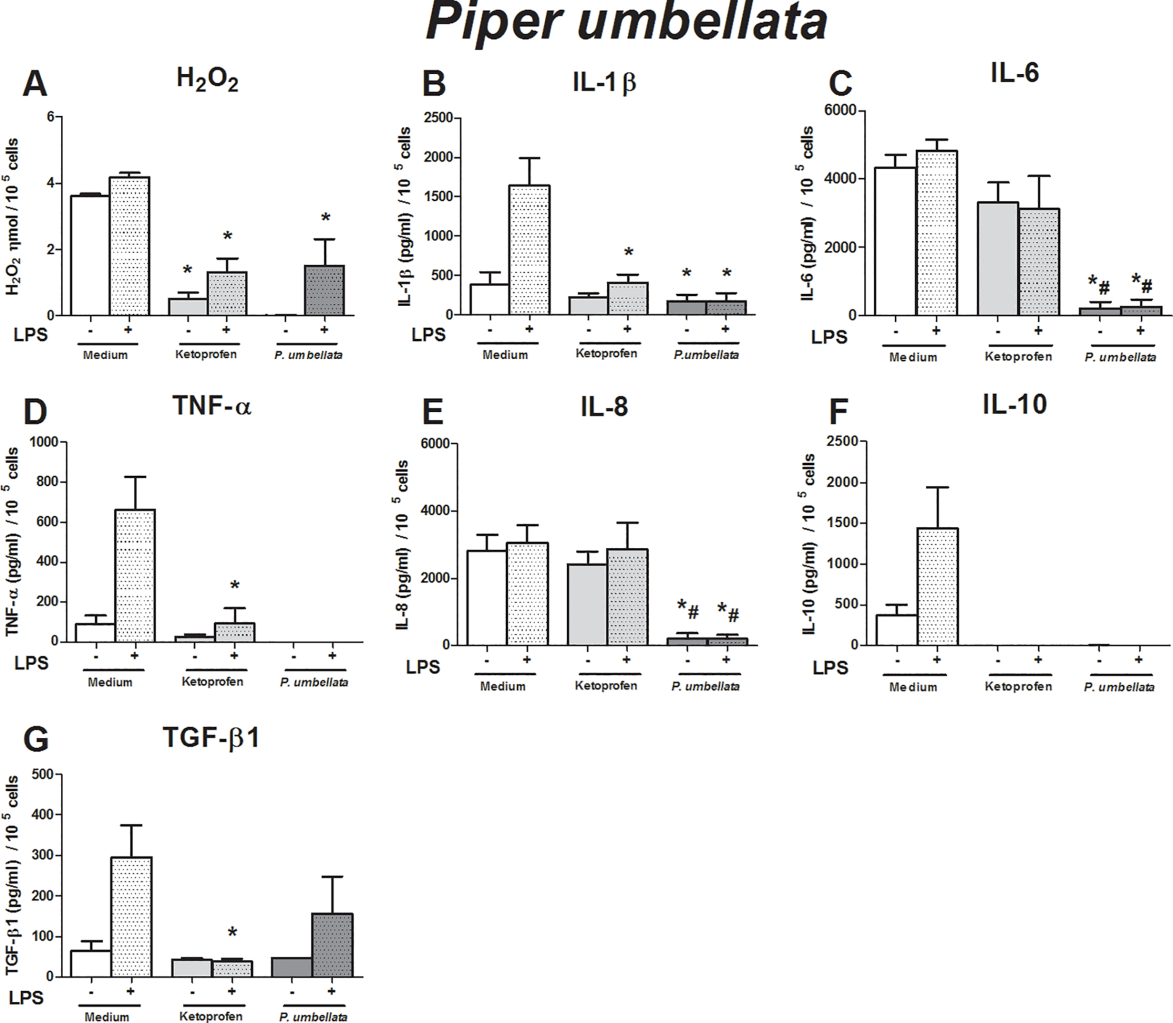
Immunomodulatory effect of crude leaf extract of *Piper umbellata* on LPS-stimulated human monocytes. The results of the controls (medium and ketoprofin, unstimulated and LPS stimulated) were used in the analyses for all extract evaluated. Data are expressed as median ± SEM (pg/mL). ANOVA with Bonferroni post-test; p < 0.05; *: significantly different from placebo treatment, #: significantly different from ketoprofen treatment.

**Fig 4:**
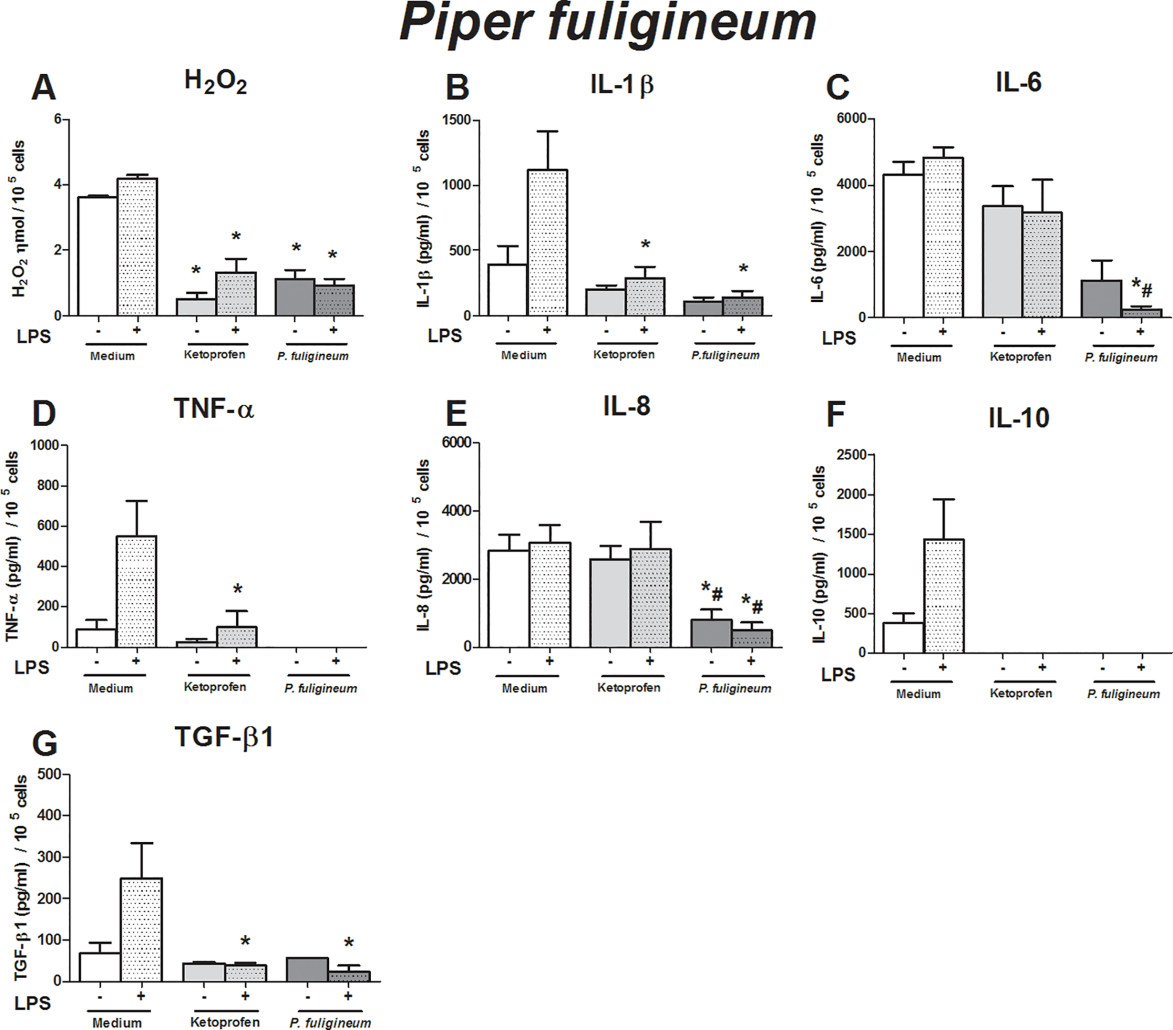
Immunomodulatory effect of crude leaf extract of *Piper fuligineum* on LPS-stimulated human monocytes. The results of the controls (medium and ketoprofin, unstimulated and LPS stimulated) were used in the analyses for all extract evaluated. Data are expressed as median ± SEM (pg/mL). ANOVA with Bonferroni post-test; p < 0.05; *: significantly different from placebo treatment, #: significantly different from ketoprofen treatment.

**Fig 5:**
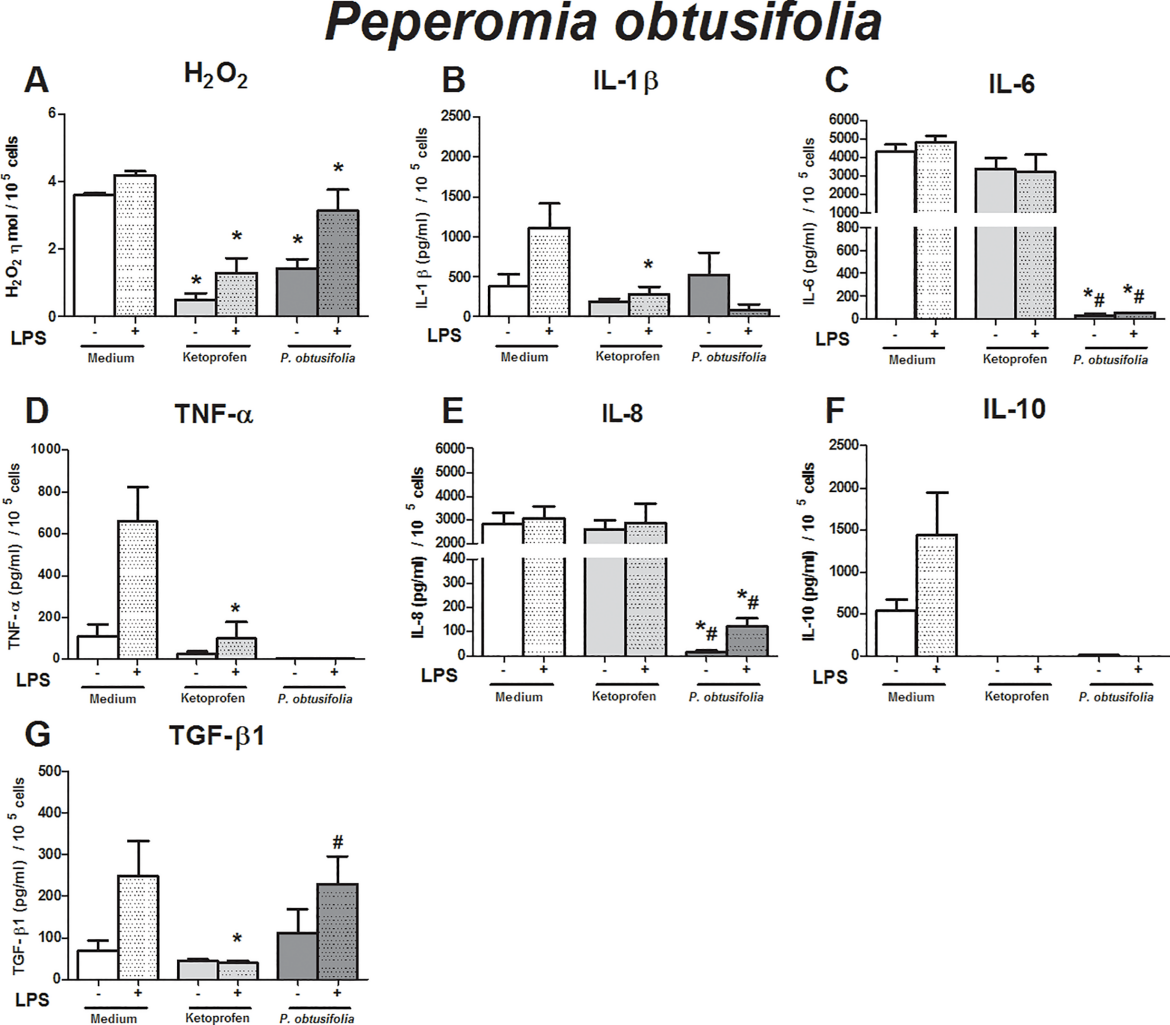
Immunomodulatory effect of crude leaf extract of *Peperomia obtusifolia* on LPS-stimulated human monocytes. The results of the controls (medium and ketoprofin, unstimulated and LPS stimulated) were used in the analyses for all extract evaluated. Data are expressed as median ± SEM (pg/mL). ANOVA with Bonferroni post-test; p < 0.05; *: significantly different from placebo treatment, #: significantly different from ketoprofen treatment.

The effects of the crude leaf extracts were evaluated using an *in vitro* inflammatory milieu triggered by LPS. As expected, the LPS-stimulated monocytes exhibited higher production of hydrogen peroxide (H_2_O_2_) (p = 0.01), IL-1β (p = 0.01), TNF-α (p = 0.016), IL-10 (p = 0.04), and TGF-β1 (p = 0.04) than non-stimulated monocytes (Figs 1–5). These findings support our *in vitro* system in determining immunomodulatory properties.

To evaluate the effects of crude leaf extract on an inflammatory milieu, we first evaluated the influence of each crude extract on human monocytes without an inflammatory stimulus, i.e., we verified if the crude extracts triggered any alteration in monocyte activity at baseline condition, and we also compared the effects of the crude extracts with those of ketoprofen. Next, using the inflammatory *in vitro* model, we compared the effects of crude leaf extracts to those of placebo treatment. Then, we compared them to those of ketoprofen treatment to identify the efficacy of crude extract over anti-inflammatory drugs.

Compared to that in unstimulated monocytes, the crude extracts of *P*. *gaudichaudianum* abrogated the production of H_2_O_2_, IL-6, TNF-α, IL-8, and IL-10 (baseline) (Fig 1). Lower production of H_2_O_2_, IL-1β, IL-6, IL-8, and TGF-β1 was observed in LPS-stimulated monocyte cell culture treated with crude extract compared to that in placebo treatment (Fig 1). The anti-inflammatory action of the crude extract was higher than ketoprofen in relation to the production of H_2_O_2_, IL-6, and IL-8 by LPS-challenged monocytes (Fig 1).

Compared to that in unstimulated monocytes, the crude extract of *P*. *arboreum* abrogated the production of IL-8 (Fig 2). Lower production of IL-1β, IL-6, IL-8, and TNF-α was observed in LPS-stimulated monocyte cell culture treated with the extracts than that observed in the placebo treatment (Fig 2). The anti-inflammatory action of the crude extract was higher than ketoprofen in relation to the production of H_2_O_2_, IL-6, IL-8, and TGF-β1 by LPS-challenged monocytes (Fig 2).

Compared to that in unstimulated monocytes, the crude extract of *P*. *umbellata* abrogated the production of H_2_O_2_, IL-1β, IL-6, TNF-α, IL-8, and IL-10 (Fig 3). Lower production of H_2_O_2_, IL-1β, IL-6, IL-8, IL-10, and TNF-α was observed in LPS-stimulated monocytes treated with the extracts than in the placebo treatment (Fig 3).

The anti-inflammatory action of the crude extract was higher than ketoprofen in relation to the production of IL-6, IL-8, and TNF-α by LPS-challenged monocytes (Fig 3).

Compared to that in unstimulated monocytes, the crude extract of *P*. *fuligineum* abrogated the production of H_2_O_2_, TNF-α, IL-8, and IL-10 (Fig 4). Lower production of H_2_O_2_, IL-1β, IL-6, IL-8, IL-10, and TNF-α was observed in LPS-stimulated monocytes treated with the extracts than in the placebo treatment (Fig 4). The anti-inflammatory action of the crude extract was higher than ketoprofen in relation to the production of IL-6, IL-8, TGF-β1, and TNF-α by LPS-challenged monocytes (Fig 4).

Compared to that in unstimulated monocytes, the crude extract of *Peperomia obtusifolia* abrogated the production of H_2_O_2_, IL-6, TNF-α, IL-8, and IL-10 (Fig 5). Lower production of H_2_O_2_, IL-6, IL-8, IL-10, TNF-α, and a tendency toward lower production of IL-1β was observed in LPS-stimulated monocytes treated with the extracts than in the placebo treatment (Fig 5). The anti-inflammatory action of the crude extract was higher than ketoprofen in relation to the production of IL-6, IL-8 TGF-β1, and TNF-α by LPS-challenged monocytes (Fig 5).

In all experimental protocols, the levels of IL-12p40 and FGF-b were below the detection limit.

## Discussion

To the best of our knowledge, we demonstrated for the first time, in this study, that all of the evaluated crude leaf extracts induced anti-inflammatory activity that was more potent than that of ketoprofen. Compared with ketoprofen, the evaluated crude extracts significantly reduced the production of crucial pro-inflammatory markers, such as IL-6, IL-8, and TNF-α. IL-8 or CXCL8, also known as neutrophil chemotactic factor, is a chemokine involved in neutrophil recruitment and degranulation during tissue injury response [40]. During inflammation, IL-6 induces T and B cell and macrophage differentiation, acute phase proteins synthesis, and T cell activation [40]. TNF-α is an important pro-inflammatory cytokine that can induce fever, apoptotic cell death, and cachexia [40]. In addition, this cytokine is commonly involved in diseases with exacerbated inflammation as a hallmark, such as rheumatoid arthritis [41], idiopathic pulmonary fibrosis (IPF) [42], Crohn’s disease [43], and type 2 diabetes [44]. These findings show that the evaluated crude extracts amplify the mechanisms underlying the anti-inflammatory response, as observed with ketoprofen.

In the present study, we observed that LPS-stimulated monocytes treated with crude extracts of *P*. *gaudichaudianum* and *P*. *fuligineum* also downregulated the production of TGF-β1 compared to LPS-stimulated untreated monocytes. TGF-β1 is a pleiotropic growth factor usually associated with anti-inflammatory and regulatory properties; however, it presents a crucial role in fibrotic diseases, including IPF [45], sarcoidosis [46], and hepatic fibrosis [47]. Infiltrating monocytes act as important sources of TGF-β1 during early fibrogenesis [48]. Thus, *P*. *gaudichaudianum* and *P*. *fuligineum* seem to present an interesting antifibrotic potential.

Reduced production of IL-1β was observed in LPS-stimulated monocytes treated with each crude extract compared to that in monocytes that were subjected to the inflammatory milieu but did not receive any treatment. This condition reinforced the anti-inflammatory potential of *P*. *gaudichaudianum*, *P*. *arboreum*, *P*. *umbellata*, *P*. *fuligineum*, and *Peperomia obtusifolia*, since IL-1β is one of the most important pro-inflammatory cytokines involved in many inflammatory/infection diseases [49,50].

Another important finding was the capacity of all extracts to prevent the production of IL-10 by monocytes, as well as by monocytes treated with ketoprofen. Usually, the production of IL-10 is increased by LPS-stimulated monocytes [51,52], probably due to an effort of cells to control the inflammation. Indeed, sepsis is associated with a monocyte hyporesponsiveness to LPS that appears to be proportional to the severity of sepsis and release of cytokines such as PGE2, TGF-β, and IL-10 [53]. Therefore, the decreased levels of IL-10 induced by the crude extracts in comparison to non-treated LPS-stimulated monocytes reinforce the idea that the compounds could better control the inflammation in our *in vitro* system.

Few studies have addressed the immune-related effects of the Piperaceae species. The ethanolic extract of the leaves of *Piper betle* Linn, which showed the accumulation of allylpyrocatechol glycosides, chavibetol glycosides, allylpyrocatechol, and chavibetol as the major chemical constituents, triggers *in vitro* downregulation of transcription of inducible nitric oxide synthase and low production of IL-12 by rat peritoneal phagocytes [48]. These studies also confirmed the immunomodulatory effect of the extract in the complete Freund’s adjuvant-induced model of arthritis in rats [54]. The aqueous extract of the aerial portion of *Peperomia pellucida* (L.) HBK had an anti-inflammatory effect in the *in vivo* model of paw edema, induced by carrageenan, thereby interfering with prostaglandin synthesis [55]. Although the studies did not characterize the extract chemically, this species is known to inhibit an important diterpene, phytol [56], which has been associated with cytotoxicity [57] and anti-histamine activity [58]. Thus, it is possible that biologically active compounds found in such extracts can trigger anti-inflammatory activity.

Monocytes are immune cells that act in numerous immunological mechanisms, such as replenishment of resident macrophages, production of dendritic cell subsets, and acute and chronic inflammatory responses [59,60]. The cellular activity of these cells involves the production of several molecules responsible for killing of pathogens (H_2_O_2_, NO), cellular recruitment (IL-8, CCL2, and CCL3), pro-inflammatory activation (TNF-α, IL-1β, and IL-6), polarization of adaptive immune response (IL-12), regulation of inflammation (IL-10 and TGF-β1), and tissue repair (TGF-β1 and bFGF) [40]. This functional plasticity is crucial for the efficiency of immune systems. On the contrary, the dysregulation of these cells is observed in several inflammatory diseases, including sepsis [61], atherosclerosis [62], rheumatoid arthritis (RA) [63], and hepatic fibrosis [47]. In general, high counts of monocytes and/or intense production of inflammatory mediators by these cells are hallmarks of these diseases [64]. High levels of TNF-α and IL-6 are associated with worse prognoses in septicemia [65] and progression and worsening of RA [66]. Furthermore, long-term use of steroid and non-steroid anti-inflammatory drugs makes treatment difficult. Therefore, in this study, we evaluated the immunotherapeutic effect of the crude extracts using the *in vitro* model of LPS-stimulated monocyte cell culture that mimics an inflammatory milieu. The principle of the assay is based on monocyte activation by LPS, the major component of the outer membrane of Gram-negative bacteria. Upon binding of LPS to the CD14 receptor and toll-like receptor 4 (TLR-4) of phagocytes [67], several transmembrane and diverse signal transduction cascades are induced to activate transcription factors of the NF-κB family [68,69]. NF-κB translocates from the cytoplasm to the nucleus where it activates target genes responsible for encoding pro-inflammatory cytokines, adhesion molecules, chemokines, growth factors, and inducible enzymes [55–57]. Although the intracellular pathways were not addressed in this study, it is possible that molecules present in the crude extract interfere with the NF-κB signaling pathway. Some anti-inflammatory agents, such as dexamethasone, prednisone, aspirin, sodium salicylate, sulindac, and sulfasalazine have been associated with the repression, or at least in part, of the NF-κB pathway [70, 71]. Thus, the *in vitro* model used in this study would be helpful in analyzing new anti-inflammatory targets, as well as the underlying mechanisms.

In conclusion, our results showed promising anti-inflammatory and antifibrotic immunomodulatory properties of crude leaf extracts of *P*. *gaudichaudianum*, *P*. *arboreum*, *P*. *umbellata*, *P*. *fuligineum*, and *Peperomia obtusifolia*, and provided insights for identification of new target drugs to enhance and/or modulate their effects and/or reduce the side-effects.

## Supporting information

S1 Fig Cytotoxic activity of crude extracts from *Piper gaudichaudianum*, *Piper umbellata*, *Piper fuligineum*, *Piper fuligineum*, and *Peperomia obtusifolia*. Peripheral blood mononuclear cells were cultured in the presence of different concentrations of the crude extracts (0.156 to 5.0 mg/mL). Concanavalin A (ConA) was used as positive control (lymphoproliferative response). Medium as added in untreated cells for control culture (CC). Data are expressed as median ± SEM (pg/mL). Repeated measures ANOVA with a Dunnett post *hoc* test; p < 0.05; * p < 0.05; *** p< 0.01; p < 0.001.
(TIF)
