# Supplementary figures and images for: Crude leaf extracts of Piperaceae species downmodulate inflammatory responses by human monocytes
Source: PLoS One. 2018 Jun 20;13(6):e0198682. doi: 10.1371/journal.pone.0198682 (PMC6010286; doi:10.1371/journal.pone.0198682)

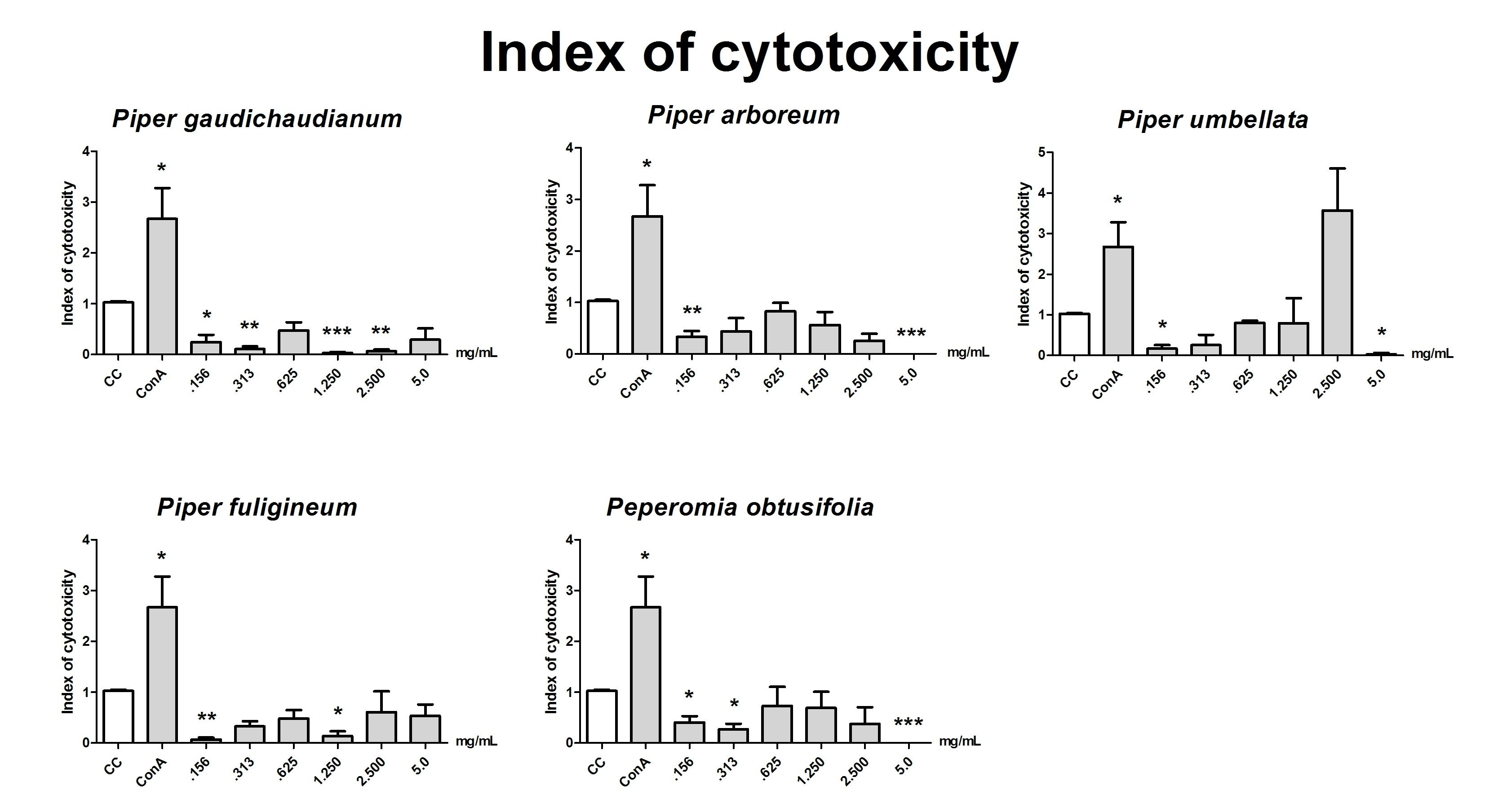

Supplement: S1 Fig — Peripheral blood mononuclear cells were cultured in the presence of different concentrations of the crude extracts (0.156 to 5.0 mg/mL). Concanavalin A (ConA) was used as positive control (lymphoproliferative response). Medium as added in untreated cells for control culture (CC). Data are expressed as median ± SEM (pg/mL). Repeated measures ANOVA with a Dunnett post hoc test; p < 0.05; * p < 0.05; *** p< 0.01; p < 0.001. (TIF) [file pone.0198682.s001.tif]
